# Protective effect of Galectin-9 in murine model of lung emphysema: Involvement of neutrophil migration and MMP-9 production

**DOI:** 10.1371/journal.pone.0180742

**Published:** 2017-07-12

**Authors:** Yuko Horio, Hidenori Ichiyasu, Keisuke Kojima, Naoki Saita, Yohei Migiyama, Toyohisa Iriki, Kazuhiko Fujii, Toshiro Niki, Mitsuomi Hirashima, Hirotsugu Kohrogi

**Affiliations:** 1 Department of Respiratory Medicine, Faculty of Life Sciences, Kumamoto University, Kumamoto, Japan; 2 Department of Immunology and Immunopathology, Kagawa University, Kagawa, Japan; Katholieke Universiteit Leuven Rega Institute for Medical Research, BELGIUM

## Abstract

**Purpose:**

Chronic obstructive pulmonary disease (COPD) is characterized by irreversible airflow obstruction and pulmonary emphysema. Persistent inflammation and remodeling of the lungs and airways result in reduced lung function and a lower quality of life. Galectin (Gal)-9 plays a crucial role as an immune modulator in various diseases. However, its role in the pathogenesis of pulmonary emphysema is unknown. This study investigates whether Gal-9 is involved in pulmonary inflammation and changes in emphysema in a porcine pancreatic elastase (PPE)-induced emphysema model.

**Materials and methods:**

Gal-9 was administered to mice subcutaneously once daily from 1 day before PPE instillation to day 5. During the development of emphysema, lung tissue and bronchoalveolar lavage fluid (BALF) were collected. Histological and cytological findings, concentrations of chemokines and matrix metalloproteinases (MMPs) in the BALF, and the influence of Gal-9 treatment on neutrophils were analyzed.

**Results:**

Gal-9 suppressed the pathological changes of PPE-induced emphysema. The mean linear intercept (Lm) of Gal-9-treated emphysema mice was significantly lower than that of PBS-treated emphysema mice (66.1 ± 3.3 μm vs. 118.8 ± 14.8 μm, respectively; p < 0.01). Gal-9 decreased the number of neutrophils and levels of MMP-9, MMP-2 and tissue inhibitor of metalloproteinases (TIMP)-1 in the BALF. The number of neutrophils in the BALF correlated significantly with MMPs levels. Interestingly, Gal-9 pretreatment *in vitro* inhibited the chemotactic activity of neutrophils and MMP-9 production from neutrophils. Furthermore, in Gal-9-deficient mice, PPE-induced emphysema progressed significantly compared with that in wild–type (WT) mice (108.7 ± 6.58 μm vs. 77.19 ± 6.97 μm, respectively; p < 0.01).

**Conclusions:**

These results suggest that Gal-9 protects PPE-induced inflammation and emphysema by inhibiting the infiltration of neutrophils and decreasing MMPs levels. Exogenous Gal-9 could be a potential therapeutic agent for COPD.

## Introduction

Chronic obstructive pulmonary disease (COPD) is currently the third leading cause of death in the world [1], and its prevalence and mortality rates are steadily increasing. Therefore, COPD is a serious health problem. Even though COPD occurs predominantly in smokers, the fact that only 15%–20% of smokers develop COPD suggests an interaction between genetic, environmental, and other factors in the etiology of COPD [2–4].

Emphysema, a major component of COPD, is defined as the abnormal enlargement of airspaces distal to the terminal bronchioles accompanied by the irreversible destruction of alveolar walls. COPD is associated with infiltrations of variable inflammatory cells including neutrophils, alveolar macrophages, and CD4+ and CD8+ lymphocytes [5–9]. The recruitment and activation of neutrophils in the lungs is particularly associated with the pathogenesis of emphysema. In addition, an imbalance in the protease–antiprotease system has long been thought to involve the destruction of alveolar walls and permanent enlargement of air spaces, resulting in emphysema [10]. Current experimental evidence shows that proteases including matrix metalloproteinase-9 (MMP-9) released from activated neutrophils and macrophages digested elastin and other structural proteins, therefore damaging alveolar units [6, 11] [9]. A recent study demonstrated that plasma levels of MMPs are associated with disease severity and are useful as biomarkers in COPD patients [12].

Based on the underlying pathophysiological mechanisms of emphysema, several potential therapeutic approaches targeting the chronic inflammation and subsequent repair have been discussed [13]. Indeed, new strategies for the treatment of COPD focus on the development of anti-inflammatory drugs, including antagonists of cytokines such as tumor necrosis factor (TNF)-α [14] and interleukin (IL)-8 [15]. However, the results of these clinical trials have been disappointing, and thus current treatments are still aimed at temporal symptomatic relief.

Gal-9 belongs to a family of 15 galectins that are characterized by their conserved carbohydrate recognition domains and their affinity for mammalian beta-galactoside [16]. Initially, Gal-9 was identified as an apoptosis inducer for thymocytes [17] and an eosinophil chemoattractant [18], playing important roles in the innate and adaptive immune responses [19]. Gal-9 is emerging as a potent immune regulator in a variety of pathological processes, including inflammation, autoimmunity, fibrosis, and cancer [20]. A recent study showed that Gal-9 down-regulates helper T type 1 (Th1) and Th17 cells responses and is related to suppression mediated by CD4+ CD25+ regulatory T (Treg) cells, mainly via interaction with the T cell immunoglobulin and domain-containing molecule 3 (Tim-3), in murine autoimmune disease models such as collagen-induced arthritis, autoimmune diabetes, and experimental autoimmune encephalomyelitis [16, 21–23]. In addition, our previous study revealed that Gal-9 regulates immune responses by expanding myeloid suppressor cells [24] and plasmacytoid dendritic cell (pDC)-like macrophages in a hypersensitivity pneumonitis mouse model and in an acute lung injury mouse model [25, 26]. However, no studies have addressed the effects of Gal-9 on an emphysema model. In the present study, we hypothesized that Gal-9 inhibits lung inflammation and attenuates emphysema in an elastase-induced emphysema model.

Some of the results of this study have been previously reported in the form of abstracts [27].

## Materials and methods

### Animals

Female C57BL/6 mice (8–10 weeks old) were obtained from Charles River Laboratories Japan (Yokohama, Japan). Gal-9–deficient (Gal-9 knock out (KO)) mice were kindly provided by GalPharma (Takamatsu, Japan). The animals were kept under specific pathogen-free conditions. In breeding rooms, we maintained a 12-hr cycle light -dark under constant temperature and humidity. All experimental protocols were approved by the Kumamoto University Animal Care and Use Committee.

### Administrations of elastase and Gal-9

All mice were anesthetized intraperitoneally (i.p.) using 0.3 mg/kg of medetomidine, 4.0 mg/kg of midazolam, and 5.0 mg/kg of butorphanol. Mouse lungs were intratracheally instilled with two units of PPE (Elastin Products, Owensville, MO) diluted in 50 μL of saline via a 24-gauge catheter on day 0. The mice were subcutaneously administered either Gal-9 (3 μg/animal) or phosphate-buffered saline (PBS) as a control, once daily for 7 days starting 1 day before PPE instillation through to day 5. Gal-9 was obtained as a recombinant human stable form according to a method described previously [28].

### Bronchoalveolar lavage and histological analysis

To obtain bronchoalveolar lavage fluid (BALF) from each group, mice were sacrificed by an overdose of anesthesia on 1, 3, 7, and 14 days after PPE inoculation. BAL was performed using a total 3 mL of PBS (3 × 1 mL). The fluid was then centrifuged at 800 × g for 5 min at 4°C and resuspended in PBS, and the cleared supernatant was collected and stored at −80°C for subsequent analyses. Total cell numbers in BALF were counted using a hemocytometer and an optimal microscope at 400× magnification. After lysating the erythrocytes, cytospin slides of BAL cells were prepared using a cytospin cytocentrifuge (Shandon Cytospin 4 Cytocentrifuge, Thermo Fisher Scientific Inc., Waltham, MA), stained with Diff-Quick solution (Sysmex), and differential cell counts were determined on at least 200 cells depend on standard morphologic criteria.

For histological analysis of lung emphysema, mice were sacrificed on day 21. The lungs were harvested in blocks, inflated by 4% buffered formalin at a constant pressure of 25 cm H_2_O, and embedded in paraffin. Histological sections (3-μm thickness) were cut for morphometric analysis. A section of lung from each group was stained with hematoxylin and eosin (H&E) [29].

The mean linear intercept (Lm) is widely accepted as an indicator of the presence of emphysema, which means the size of air space. Lm and alveoli number were calculated as previously described [30]. In brief, Lm was measured by dividing the total length of line drawn across the 20 randomly selected lung fields by number of intercepts that the lines with alveolar septum at 200× magnification. Alveoli number was determined by the number of measurements made for the Lm.

### Measuring cytokines and matrix metalloproteinases

To measure levels of cytokines and matrixmetalloproteinases (MMPs), the concentrations of keratinocyte-derived cytokine (KC), macrophage inflammatory protein-2 (MIP-2), lipopolysaccharide (LPS)-induced CXC chemokine (LIX), which are murine neutrophil chemoattractants, MMP-9, MMP-2 and tissue inhibitor of metalloproteinases (TIMP)-1 in BALF were determined by enzyme-linked immunosorbent assay (ELISA) using cytokine-specific kits (R&D Systems, Minneapolis, MN) according to the manufacturer’s instructions.

### Assay for chemotactic activity

Neutrophil chemotaxis was examined using a 48-well chemotaxis chamber (Neuro Probe, Gaithersburg, MD) with a polyvynilpyrrolidone-free 3-μm nucleopore filter as described [25]. To isolate neutrophils for the assay of chemotactic activity, mice were injected i.p. with 3 mL of sterile 3% Brewer Thioglycollate medium [31]. Five hours later, cells were harvested by peritoneal lavage. This procedure routinely yielded 20-million total cells when pooled, with 97% of the cells as neutrophils. Neutrophils were suspended in Ca^2+^- and Mg^2+^-free Hanks’ balanced salt solution (HBSS) and preincubated with 1–100 nM Gal-9 or PBS for 4 h [32]. Thereafter, the treated neutrophils were centrifuged at 800 × g for 8 min and washed with HBSS. The neutrophils (5 × 10^4^ cells/well) were pre-incubated with HBSS and then placed in the upper chemotaxis chamber and the medium containing either KC (20 ng/mL) (R&D Systems, Minneapolis, MN) or PBS was poured into the lower chamber. To inhibit the effect of Gal-9, neutrophils were pre-incubated with both 1–100 nM Gal-9 and 5 nM lactose, a representative beta-galactoside, or PBS for 4 h, and the chemotactic activity was examined.

The chamber was incubated at 37°C in a 5% CO_2_-containing atmosphere for 1 h. When the incubation period was complete, the filter was carefully removed. Neutrophils that did not migrate were wiped off the upper surface of the filter against a rubber blade (Neuro Probe). The remaining neutrophils on filter were counted after Diff-Quick staining as previously reported [25]. Chemotactic activity was expressed as the number of migrated neutrophils per high-powered field magnified at 400×.

### MMP-9 production of neutrophils obtained from Gal-9-treated mice

To investigate the effect of Gal-9 in MMP-9 secretion by neutrophils, C57BL/6 mice were subcutaneously administered either Gal-9 (3 μg/animal) or PBS as a control at 0h and 24h, then mice were injected i.p. with 3 mL of sterile 3% Brewer Thioglycollate medium to induce neutrophils. Five hours later, cells were harvested by peritoneal lavage. The neutrophils were washed and resuspended in RPMI-1640 medium. Then a total 1.0 × 10^6^ neutrophils in 1mL were incubated in 24-well plates at 37°C in a 5% CO_2_-containing atmosphere for 1 h. After incubation, neutrophils were centrifuged for at 800 × g for 5 min and the supernatant was collected for the measurement of MMP-9 by ELISA.

### Statistical analysis

Data are presented as the mean ± standard of the mean (SEM). Statistical analyses were performed by one-way ANOVA, followed by post-hoc analysis with the Tukey-Kramer test and Mann-Whitney test. Correlations between variables were assessed using nonparametric two tailed Spearman rank correlation coefficients. A p value of less than 0.05 was used as a threshold for statistical significance. All statistical analyses were performed using Prism 5 software (GraphPad Software, San Diego, CA).

## Results

### Gal-9 attenuates the PPE-induced emphysema

**Fig 1:**
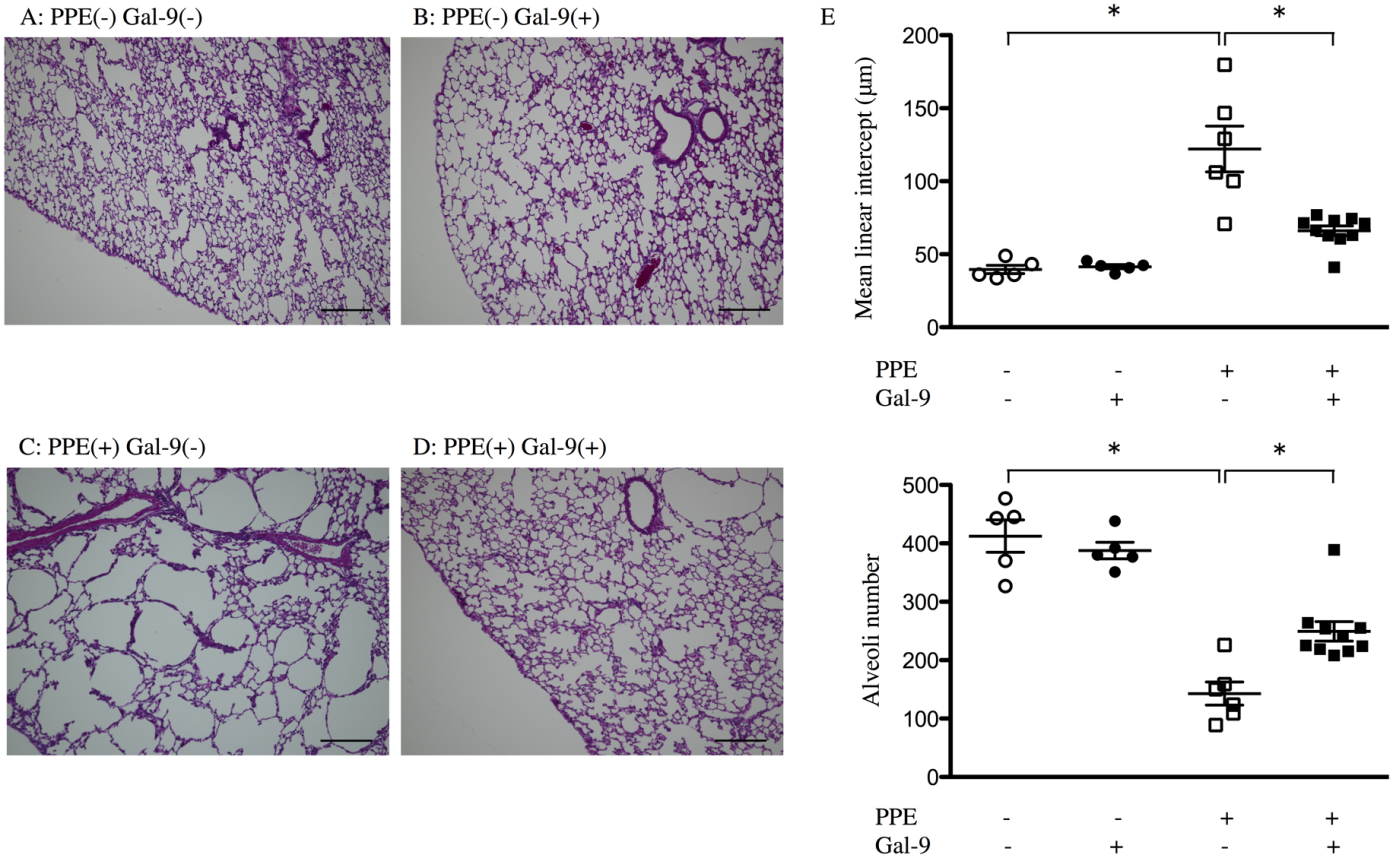
Gal-9 attenuates pulmonary emphysema induced by PPE. Mice were given 2 units of PPE in saline or saline alone intratracheally on day 0. PPE- or saline-treated mice were further subdivided and treated subcutaneously with Gal-9 in PBS or PBS alone once a day from one day before PPE instillation to day 5. Lungs were resected on day 21 and stained with hematoxylin and eosin (H&E) for morphometric analysis. Emphysema was severe in PPE (+) Gal-9 (-) (C) compared to that in PPE (-) Gal-9 (-) (A) and PPE (-) Gal-9 (+) (B), and emphysema was attenuated in PPE (+) Gal-9 (+) (D). Scale bars = 200 μm. Lm was significantly increased in PPE (+) Gal-9 (-) (n = 6) compared to that in PPE (-) Gal-9 (-) (n = 5) and PPE (-) Gal-9 (+) (n = 5) and was significantly decreased in PPE (+) Gal-9 (+) (n = 10) compared to that in PPE (+) Gal-9 (-) (E, upper panel). The number of alveoli was significantly lower in PPE (+) Gal-9 (-) (n = 6) than in PPE (-) Gal-9 (-) (n = 5), and PPE (-) Gal-9 (+) (n = 5) and was significantly higher in PPE (+) Gal-9 (+) (n = 10) (E, lower panel). Data are presented as the mean ± SEM. *p < 0.05. Abbreviations: PPE, porcine pancreatic elastase; Gal-9, galectin-9; PBS, phosphate buffered saline; Lm, mean linear intercept; SEM, standard error of the mean.

Gal-9 attenuated the emphysema induced by PPE. Histological analysis on day 21 revealed that PPE-treated mice that were administered PBS (PPE-treated control mice) showed diffuse emphysema lesions, as indicated by alveolar wall destruction and marked enlargement of airspace compared with saline-treated mice (non-PPE-treated mice), but Gal-9 administration did not affect lung structure in non-PPE-treated mice (Fig 1A–1C). The mean linear intercept (Lm) was markedly increased in PPE-treated control mice (118.8 ± 14.8 μm; n = 6) as compared to the non-PPE-treated mice (39.56 ± 2.85 μm; n = 5). By contrast, PPE-treated mice with exogenously added Gal-9 showed significant inhibition of emphysema (Fig 1D) and a significantly smaller Lm (66.07 ± 3.27 μm; n = 10) than that of PPE-treated control mice, with a significant increase in alveoli number (Fig 1E).

### Gal-9 prevents PPE-induced neutrophil and lymphocyte infiltrations in BALF

**Fig 2:**
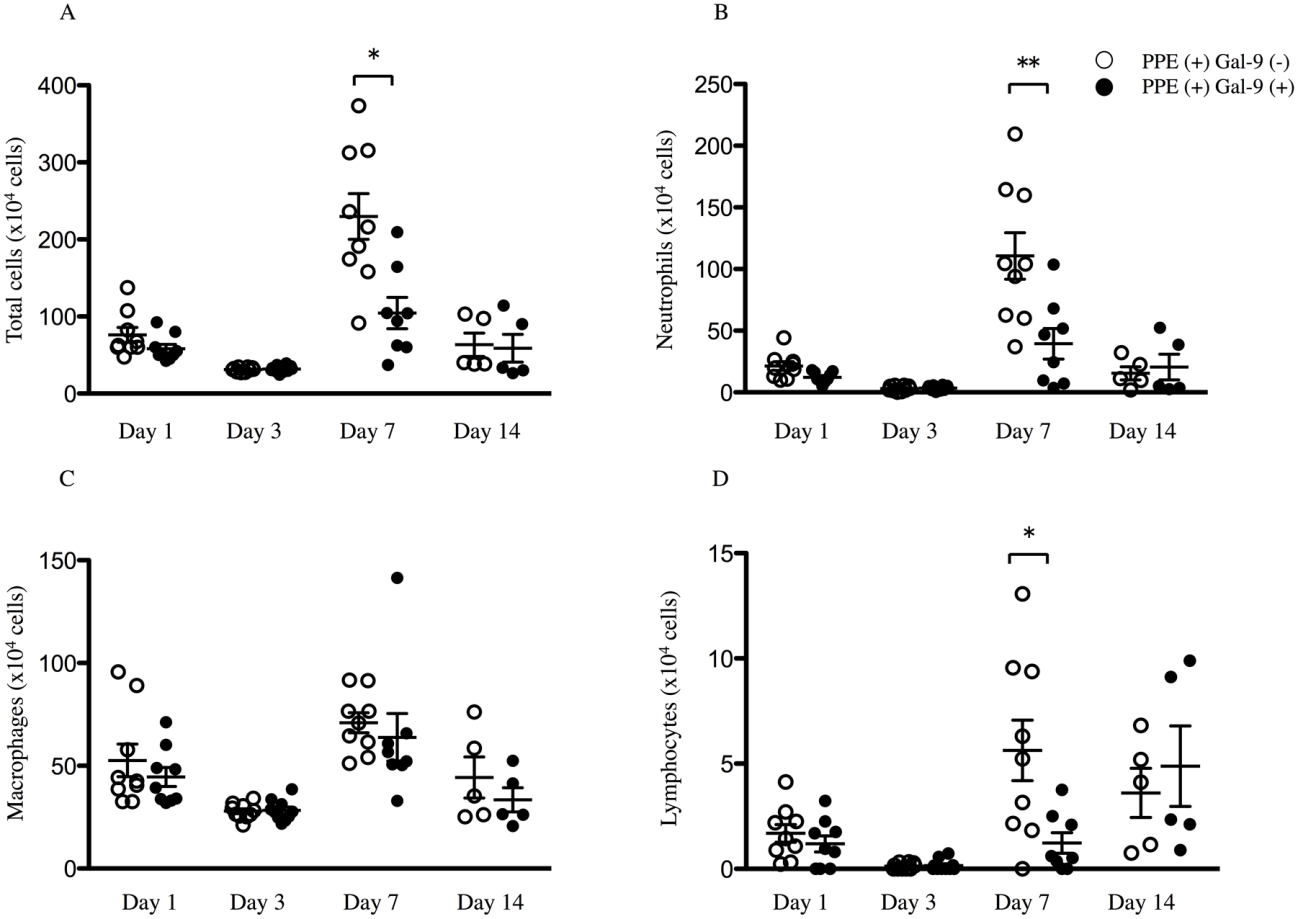
Gal-9 prevents PPE-induced neutrophil and lymphocyte inflammation in BALF. PPE-treated mice were given either Gal-9 in PBS or PBS alone from 1 day before PPE instillation to day 5, and BALF was collected on days 1, 3, 7, and 14. The number of total cells was counted, and cell differentiation was determined (n = 5–10). The number of total cells was high on day 7 in PPE (+) Gal-9 (-) (open circle) and the number of total cells in PPE (+) Gal-9 (+) (crosed circle) on day 7 was significantly lower than that of in PPE (+) Gal-9 (-) (A). Similarly, neutrophils and lymphocytes were significantly lower in PPE (+) Gal-9 (+) on day 7 than in PPE (+) Gal-9 (-) (B and D), and macrophages were not significantly difference between in PPE (+) Gal-9 (+) and in PPE (+) Gal-9 (-) (C). Data are presented as the mean ± SEM. *p < 0.05, **p < 0.01. Abbreviations: BALF, bronchoalveolar lavage fluid; PPE, porcine pancreatic elastase; PBS, phosphate buffered saline; Gal-9, galectin-9; SEM, standard error of the mean.

PPE-treated mice showed marked leukocyte infiltration into the bronchoalveolar space preceding the development of emphysema. As shown in Fig 2, the total number of BAL cells transiently increased on day 7 and returned to basal levels on day 14 (Fig 2A). Differential cell analyses showed that the numbers of neutrophils and lymphocytes markedly increased on day 7, and the exogenous administration of Gal-9 in the PPE-induced emphysema model resulted in a striking decrease in the number of total cells, neutrophils, and lymphocytes, with a 54.5%, 74.3%, and 78.2% reduction, respectively, compared to controls (Fig 2A, 2B and 2D). There were no significant differences in the number of macrophages between the experimental groups (Fig 2C) and eosinophils were not detected in both experimental groups.

### Gal-9 reduces MMP-9, MMP-2 and TIMP-1 but not chemokines in BALF

**Fig 3:**
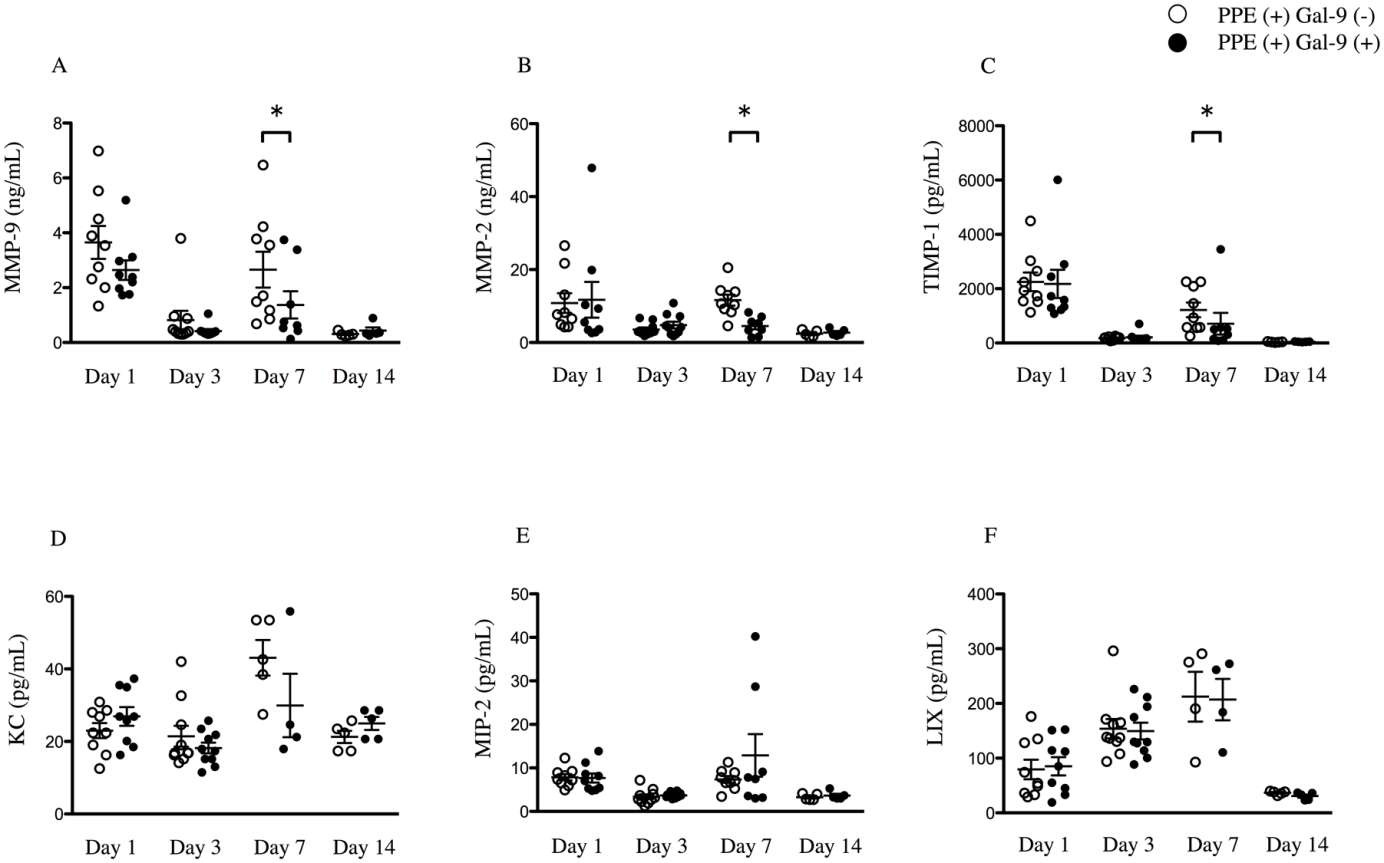
Gal-9 treatment decreases levels of MMP-9, MMP-2 and TIMP-1 but not neutrophil chemoattractants in BALF. PPE-treated mice were given either Gal-9 in PBS or PBS alone from 1 day before PPE instillation to day 5. BALF was collected on days 1, 3, 7, and 14, and the levels of MMP-9, MMP-2, TIMP-1, KC, MIP-2, and LIX protein were determined (A–F; n = 4–10). On day 7, the levels of MMP-9, MMP-2, TIMP-1 was significantly lower in PPE (+) Gal-9 (+) (closed circle) than in PPE (+) Gal-9 (-) (open circle) (A-C), but the levels of KC, MIP-2, and LIX did not differ (D-F). Data are presented as the mean ± SEM. *p < 0.05. Abbreviations: Gal-9, galectin-9; MMP-9, matrix metalloproteinase-9; MMP-2, matrix metalloproteinase-2; TIMP-1, tissue inhibitor of metalloproteinases-1; BALF, bronchoalveolar lavage fluid; KC, keratinocyte-derived cytokine; MIP-2, macrophage inflammatory protein-2; LIX, lipopolysaccharide-induced CXC chemokine; PPE, porcine pancreatic elastase; PBS, phosphate buffered saline; SEM, standard error of the mean.

To assess the impact of exogenous Gal-9 administration on MMPs and TIMP-1 production, the levels of MMP-9, MMP-2 and TIMP-1 in BALF were compared between the Gal-9-treated and control mice. Gal-9 significantly reduced MMP-9, MMP-2 and TIMP-1 levels in BALF on day 7 in the emphysema model (Fig 3A–3C). To further investigate the influence on the expression of chemokine for neutrophils, we measured levels of KC, MIP-2, and LIX in the BALF on day 7. No differences in the levels of these chemokines were observed between the experimental groups (Fig 3D–3F). These results suggest that Gal-9 attenuates PPE-induced emphysema by suppressing neutrophil recruitment and MMP-9 and MMP-2 production but does not reduce the levels of chemoattractants for neutrophils.

**Fig 4:**
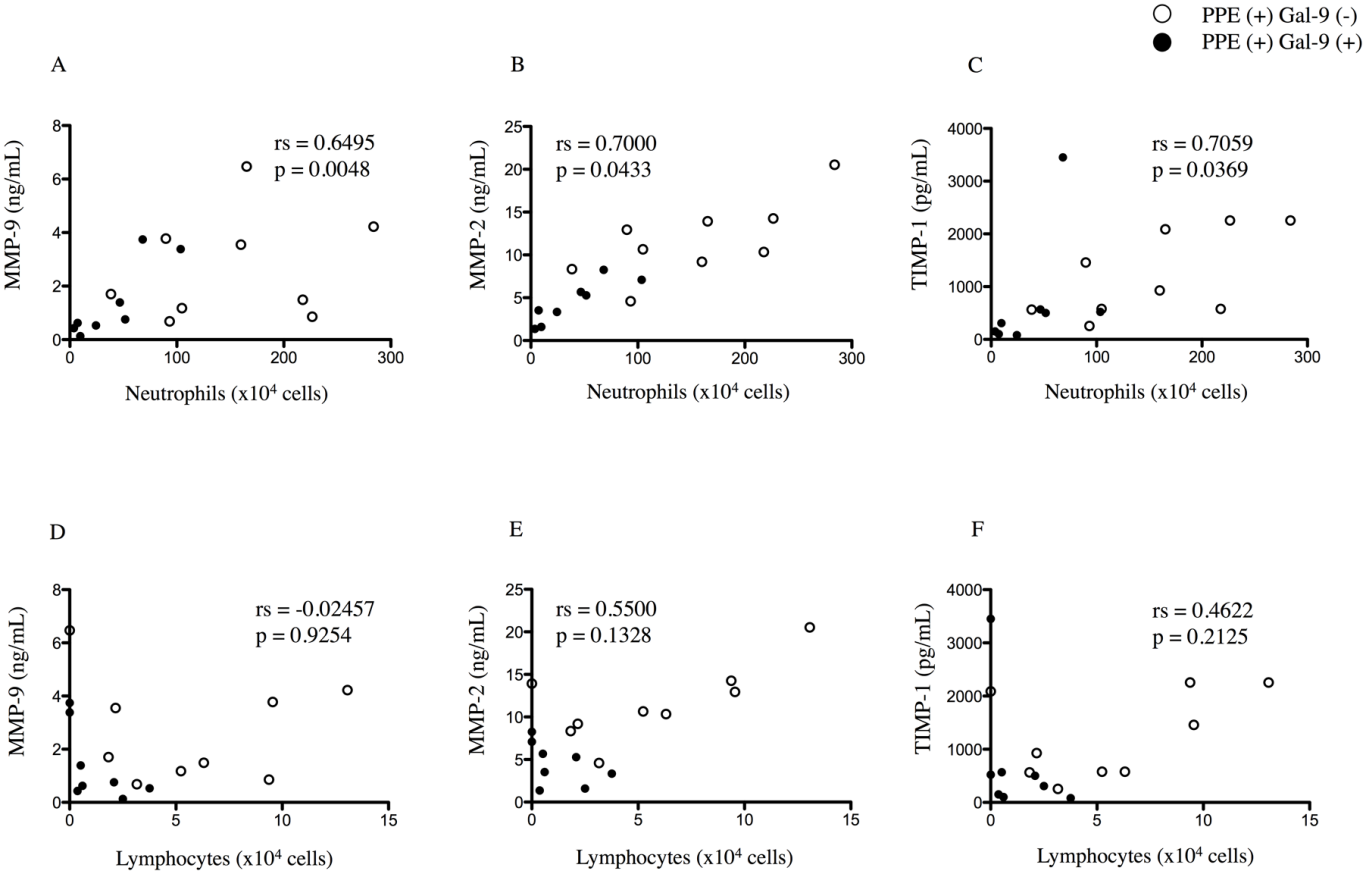
Numbers of neutrophils correlates with MMP-9, MMP-2 and TIMP-1 levels in BALF. Correlations between the levels of MMP-9, MMP-2, TIMP-1 and the cell fractions of BALF in PPE (+) Gal-9 (-) (n = 9) (open circle) and PPE (+) Gal-9 (+) (n = 8) (closed circle) on day 7 were analyzed using Spearman’s correlation analysis. Significant positive correlations were observed between the levels of MMP-9, MMP-2, TIMP-1 and the numbers of neutrophils (A-C) but not the number of lymphocytes (D-F) in BALF. Abbreviations: MMP-9, matrix metalloproteinase-9; MMP-2, matrix metalloproteinase-2; TIMP-1, tissue inhibitor of metalloproteinases-1; Gal-9, galectin-9; BALF, bronchoalveolar lavage fluid; rs; Spearman’s rank correlation coefficient.

Interestingly, the MMP-9, MMP-2 and TIMP-1 levels in BALF correlated significantly with the number of neutrophils (rs = 0.6495; p = 0.0048, rs = 0.7000; p = 0.0433, rs = 0.7059; p = 0.0369, respectively) (Fig 4A–4C) but not the number of lymphocytes (rs = -0.02457; p = 0.9254, rs = 0.5500; p = 0.1328, rs = 0.4622; p = 0.2125, respectively) (Fig 4D–4F).

### Gal-9 inhibits chemotaxis of neutrophils *in vitro*

**Fig 5:**
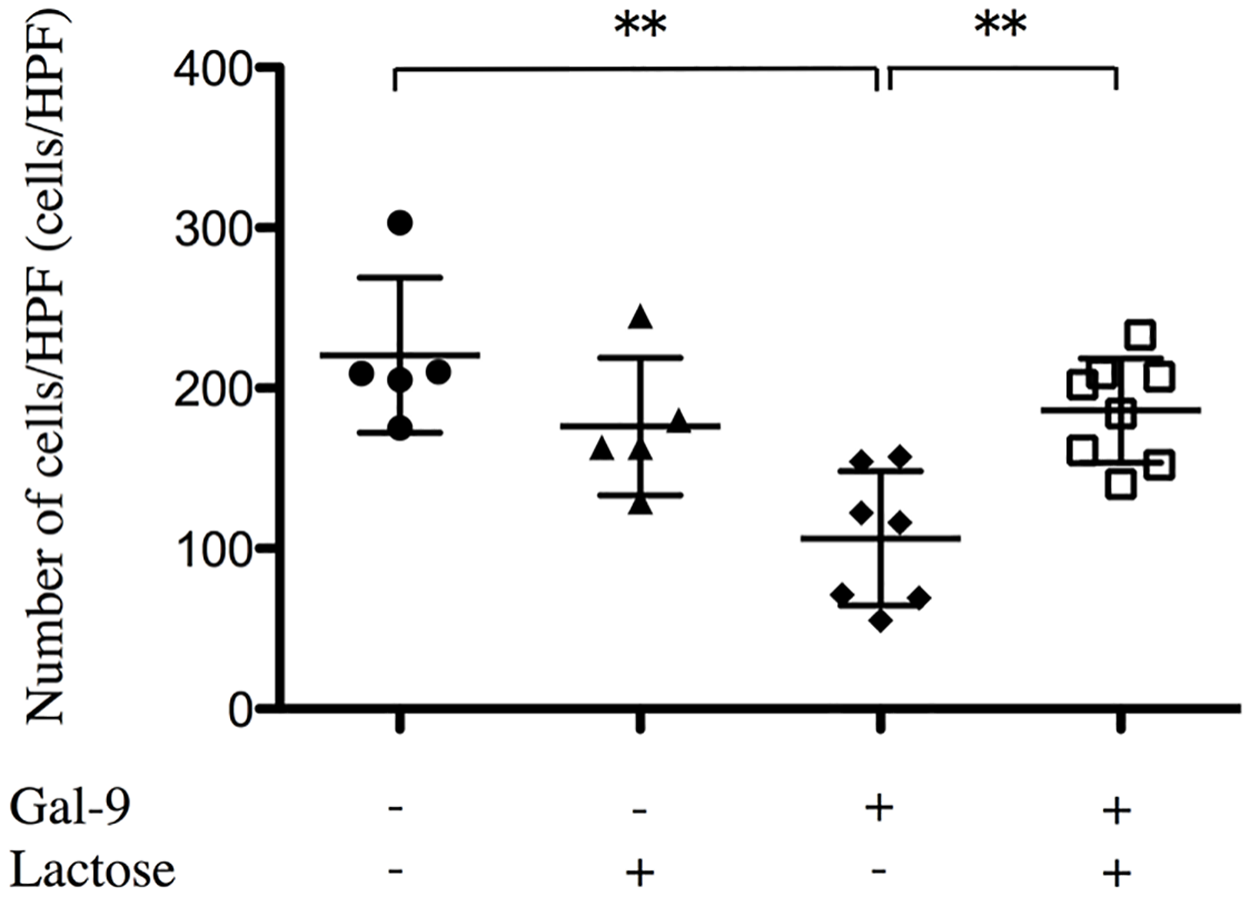
Gal-9 inhibits neutrophil chemotaxis, an effect that is reversed by the addition of lactose. The effect of 4-h preincubation of 10-nM Gal-9 (closed bar) or HBSS (open bar) as a control on neutrophil chemotaxis in response to KC (20 ng/mL) was examined in 48-well chemotaxis chambers. Gal-9 significantly reduced chemotaxis and the addition of lactose reversed this chemotaxis inhibition. Data are representative and are represented as the mean neutrophil count per high-powered field at 400× magnification (Number of cells/HPF) ± SEM. n = 5–8. *p < 0.05; **p < 0.01. Abbreviations: Gal-9, galectin-9; HBSS, Hanks’ balanced salt solution; KC, keratinocyte-derived cytokine; HPF, high power field; SEM, standard error of the mean.

Because Gal-9 appeared to modulate neutrophil recruitment rather than inhibiting chemokine production, we examined whether Gal-9 directly inhibits the chemotactic activity of neutrophils toward the chemokine KC. Gal-9 had no effect on cell viability as confirmed by trypan blue staining. The chemotactic activity of Gal-9–preincubated neutrophils was significantly decreased, and the effect was reversed by the addition of lactose to Gal-9 (Fig 5). These results suggested that Gal-9 directly suppresses KC-induced neutrophil chemotaxis.

### Gal-9 inhibits the MMP-9 secretion from neutrophils

**Fig 6:**
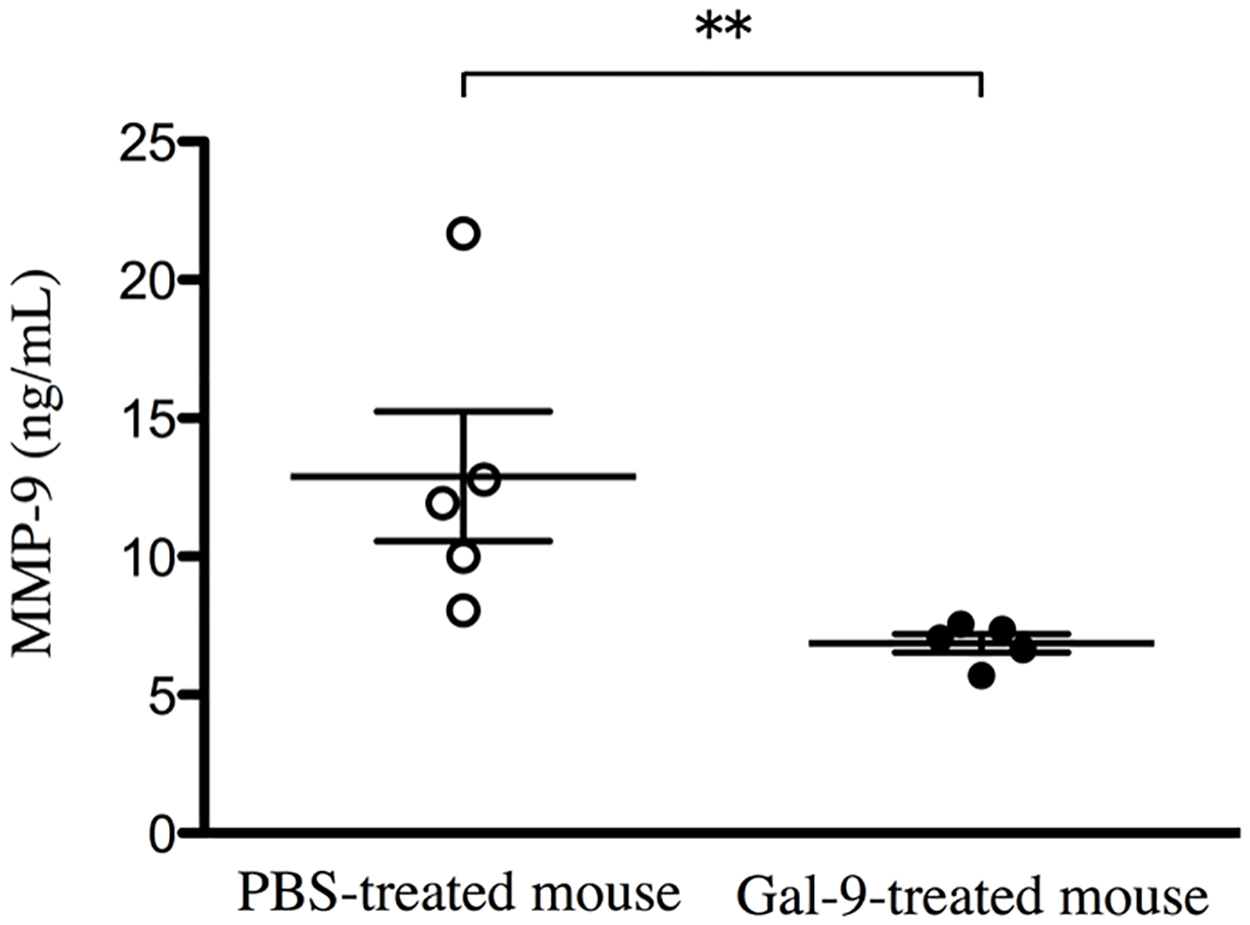
Gal-9 inhibits the MMP-9 secretion from neutrophils. Mice were subcutaneously administered either Gal-9 (3 μg/animal) or PBS alone at 0h and 24h, then thioglycollate medium was injected intraperitoneally to induce neutrophils. Five hours later, neutrophils were harvested by peritoneal lavage and incubated for 1 h. The level of MMP-9 in culture supernatant was significantly lower in Gal-9-treated mice than in PBS-treated mice (n = 5 per each group). Data are presented as the mean ± SEM. *p < 0.05. Abbreviations: Gal-9, galectin-9; PBS, phosphate buffered saline; MMP-9, matrix metalloproteinase-9; SEM, standard error of the mean.

To investigate the effect of Gal-9 in MMP-9 secretion from neutrophils, the neutrophils obtained from PBS-treated mice or Gal-9-treated mice was cultured in vitro for 1 h. The level of MMP-9 in culture supernatant of neutrophils obtained from Gal-9-treated mice were significantly lower than that from PBS-treated mice (Fig 6). These results suggest that Gal-9 inhibits the MMP-9 secretion from neutrophils and reduction of MMP-9 itself might affect the migration of neutrophils.

### PPE-induced emphysema is exacerbated in Gal-9-deficient mice

Previous research has demonstrated that Gal-9 in the lung maintains cell and tissue homeostasis under physiological conditions and after inflammatory responses [33]. To clarify whether endogenous Gal-9 has protective effects on the development of emphysema, we examined the histological and cellular changes of PPE-induced emphysema in Gal-9-deficient mice.

**Fig 7:**
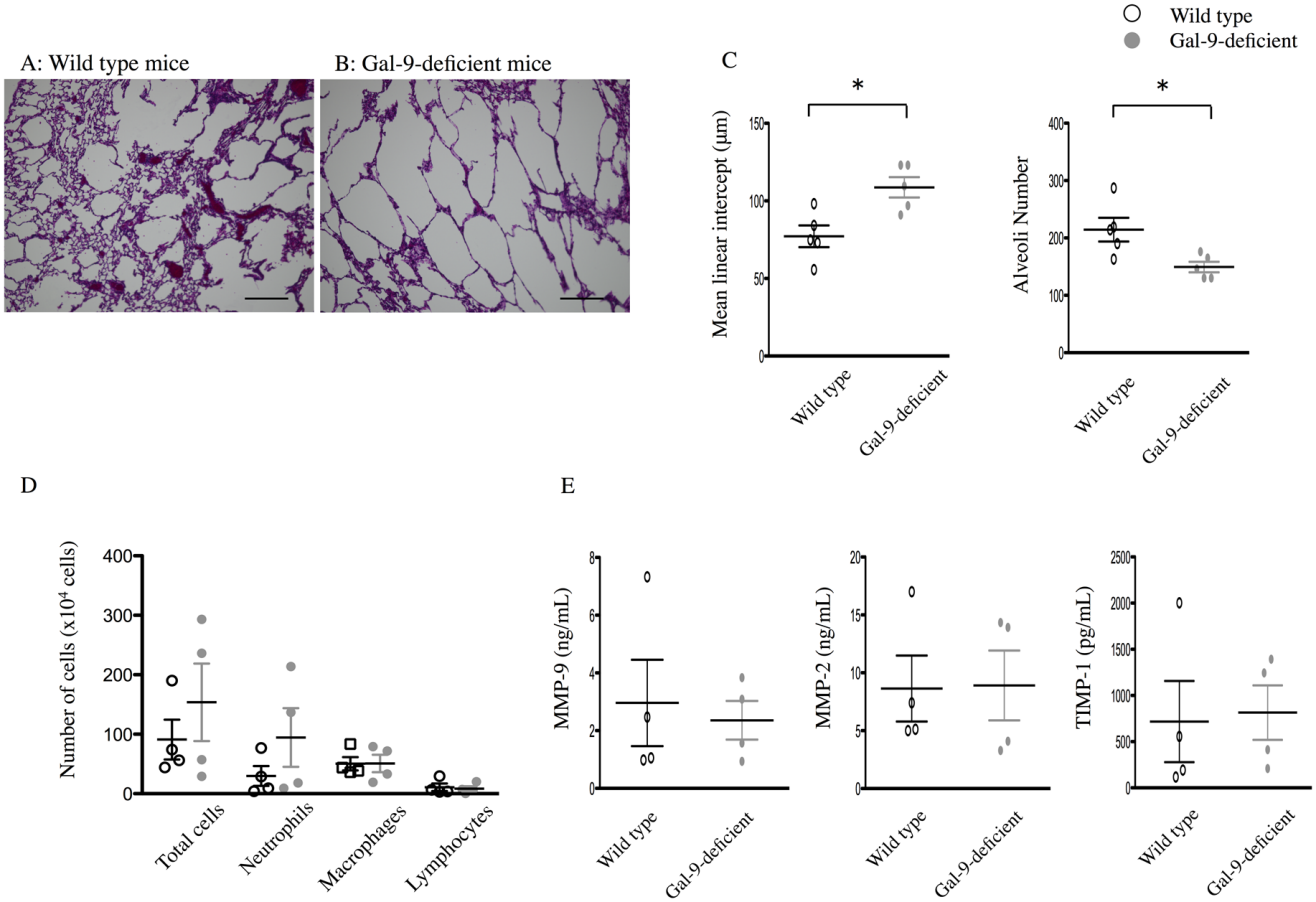
PPE-induced lung emphysema is exacerbated in Gal-9–deficient mice. Mice were instilled 2 units of PPE in saline or saline alone intratracheally on day 0. The lungs were resected on day 21, and morphometric analysis was performed. Emphysematous changes in Gal-9-deficient mice were increased compared to those in WT mice (H&E stain) (A and B), and Lm was significantly higher in Gal-9–deficient mice (gray circle) compared to that in WT mice (open circle), with a significant decrease in alveoli number (n = 5 per group) (C). Scale bars = 200 μm. Numbers of total cells and neutrophils in BALF on day 7 were greater in WT mice and in Gal-9-deficient mice, and higher in Gal-9-deficient mice than in WT mice, but the difference was not statistically significant (n = 4 per group) (D). Numbers of lymphocytes and macrophages and the levels of MMP-9, MMP-2 and TIMP-1 did not differ between WT mice and Gal-9-deficient mice (n = 4 per group) (D and E). Data are presented as the mean ± SEM. *p < 0.05. Abbreviations: PPE, porcine pancreatic elastase; Gal-9, galectin-9; WT, wild–type mice; Lm, mean linear intercept; BALF, bronchoalveolar lavage fluid; MMP-9, matrix metalloproteinase-9; MMP-2, matrix metalloproteinase-2; TIMP-1, tissue inhibitor of metalloproteinases-1; SEM, standard error of the mean.

At day 21 of PPE administration, air space enlargements and alveolar wall destruction were more severe in Gal-9-deficient mice than in WT mice (Fig 7A and 7B). Lm values for Gal-9-deficient mice (108.7 ± 6.58 μm; n = 5) were significantly increased compared to WT mice (77.19 ± 6.97 μm; n = 5) 21 days after treatment with PPE (Fig 7C). The number of total cells and neutrophils in BALF on day 7 was higher in Gal-9-deficient than in WT mice, although this difference was not statistically significant. In contrast, the number of lymphocytes and macrophages were not different (Fig 7D). The levels of MMP-9, MMP-2 and TIMP-1 did not differ between WT mice and Gal-9-deficient mice 7 days after PPE treatment (Fig 7E). These histological and cellular evaluations demonstrate that Gal-9-deficient mice are more susceptible to PPE-induced changes in the lung and that endogenously-released Gal-9 has inhibitory effects on PPE-induced emphysema.

## Discussion

In this study, we addressed the contribution of Gal-9 to the pathogenesis of emphysema. We found that Gal-9 attenuated PPE-induced emphysema in a murine model by inhibiting the infiltration of neutrophils and MMP-9 production in the lung. Gal-9 treatment inhibited the neutrophil chemotaxis in response to chemoattractant. In Gal-9 deficient mice, PPE-induced pulmonary emphysema was exacerbated by the enhanced neutrophil recruitment in the lung.

COPD is a major worldwide public health concern as it leads to a slowly-progressive deterioration in pulmonary function, substantial morbidity, and increased mortality [1]. One of the current accepted hypotheses regarding the pathogenesis of cigarette-smoke-induced emphysema is the involvement of a protease–antiprotease imbalance. Based on this hypothesis, experimental emphysema mice models were designed by the administration of elastolytic enzymes, such as PPE, either by intratracheal instillation or aerosol inhalation [34, 35]. The PPE-induced emphysema models have been used in studies for more than 40 years [36] to reproduce some of the characteristics of cigarette-smoke-induced disease in humans, such as enlargement of air spaces, inflammatory cell influx into the lung, and systemic inflammation [37]. Using this model, several studies have developed new pharmacological strategies for attenuating the inflammation or remodeling processes. Although implicated in health and disease, a detailed understanding of the involvement of galectins in pulmonary emphysema is lacking. In the present study, we demonstrated that treatment with Gal-9 attenuates PPE-induced emphysema (Fig 1).

Gal-9-treated animals had lower numbers of total cells, neutrophils, lymphocytes in BALF (Fig 2A, 2B and 2D), suggesting that Gal-9 had an important anti-inflammatory effect in this model. Gal-9 effectively reduced the cell fraction of neutrophils in BALF from 69% to 35%. One of the pathogenesis of emphysema and COPD is pulmonary inflammation, and neutrophil recruitment into the lung is thought to play a central role in its development. Additionally, previous data in animal models have indicated a strong relationship between inflammation and emphysematous changes [38].

The anti-inflammatory effects of Gal-9 in the lung were previously reported in the hypersensitivity pneumonitis mouse model [24] and LPS-induced acute lung injury model that we reported [25]. Most reports show that Gal-9 has effects reducing the levels of pro-inflammatory cytokines and chemotactic factors interferon-γ (IFN-γ), IL-1β, IL-17, IL-6, KC, TNF-α, and monocyte chemoattractant protein-1 (MCP-1) in the lung. Even though KC, MIP-2, and LIX are the main signaling molecules in the recruitment of neutrophils and macrophages, Gal-9 did not reduce their levels in BALF during the process of emphysema formation (Fig 3D–3F). In this PPE-induced emphysema model, the levels of chemokines in BALF were not elevated as compared to other acute inflammatory mice models [16], including our LPS-induced acute lung injury model [25]. One reason for this lack of change in chemokine expression is that the functions of chemokine-producing cells such as macrophages and endothelial cells are likely to be modulated by the administration of Gal-9. Arikawa *et al*. reported that treatment with Gal-9 in an arthritis model repressed macrophage activity, resulting in a significant reduction in proinflammatory cytokine expression and up-regulation of the anti-inflammatory cytokine IL-10 [39]. They also showed that the number of CD11b^+^Ly-6C^high^ immunosuppressive macrophages was increased by treatment with Gal-9 in experimental lung inflammation [24].

Similarly, our previous study using a mouse model of acute lung injury demonstrated that Gal-9 treatment increased the number of CD14^-^ plasmacytoid dendritic cell-like macrophages and suppressed the accumulation of neutrophils in the lung by decreasing macrophage-derived chemokines and neutrophil-activating pro-inflammatory cytokines [25]. Therefore, in the present study, we examined the influence of these suppressor macrophages in a Gal-9–treated emphysema model. However, the number of these cells present in BALF were unaffected by exogenously added Gal-9 (data not shown). Further studies are required to more directly address the anti-inflammatory effects of Gal-9 during emphysema pathogenesis.

We examined whether Gal-9 inhibits the migration of neutrophils in response to chemotactic stimulation with KC. In our *in vitro* study, Gal-9 treatment significantly reduced the chemotactic activity of neutrophils, and the effect was reversed by lactose, a representative beta-galactoside (Fig 5). Thus, the inhibition of chemotaxis by Gal-9 may be dependent on its carbohydrate moieties and seems to involve direct interaction between Gal-9 and neutrophils. However, this finding seems to conflict with previous findings indicating the role of Gal-9 as an activator of neutrophils [40][41]. Steichen *et al*. demonstrated that Gal-9 induces the production of inflammatory mediators by neutrophils in a bacterial septic model and that the survival of the Gal-9 blockade was significantly greater than in WT mice [40]. Moreover, a recent study showed that Gal-9 directly binds to TIM-3 on neutrophils and up-regulates neutrophil functions including phagocytosis, protease degranulation, and inflammatory infiltration [41]. Conversely, the mechanism underlying the inhibitory effect of Gal-9 on neutrophil migration is not fully understood. This inhibition of chemotaxis by Gal-9 is similar to that observed by Gal-1 and -3, which also inhibit chemotaxis and the transendothelial migration of neutrophils [42, 43]. The effects of galectins are complex and vary depending on the local concentration, differentiation status of the target cells, and the type of inflammatory condition [33]. Further studies are required to determine the precise underlying mechanisms.

In the present study, Gal-9 was observed to decrease the levels of MMP-9, MMP-2 and TIMP-1 in BALF (Fig 3A–3C). In an animal model of emphysema and in patients with COPD, several MMPs, including MMP-9, are elevated in sputum, BALF, and lung tissue specimens [2, 30, 44, 45] and are related to poor lung function. MMP inhibition attenuates emphysema and small-airway remodeling in guinea pigs and hamsters [46, 47]. However, D’Armiento *et al*. found no significant correlation between MMP-9 and emphysema severity [48]. Zheng *et al*. reported that MMP-2 might also be important in other mouse models of pulmonary emphysema [49]. But together with MMP-9, MMP-2 degrades elastin and type IV collagen, the major component of lung structures. MMP-2 also may affect the induction of emphysema and alveolar wall damage. Gal-9 expression is regulated by MMPs other than MMP-2 and -9, and Gal-9 contains MMP cleavage sites [50] Although little is known about the involvement of Gal-9 in MMP-9 expression, one study revealed that infected Gal-9–deficient mice displayed significantly reduced levels of MMP-9 in lung extracts and accompanying decreases in neutrophil infiltration and inflammatory mediators [40]. In the present study, exogenous administration of Gal-9 in an emphysema animal model reduced the levels of MMP-9, MMP-2 and TIMP-1 in BALF (Fig 3A–3C), and these MMPs levels correlated significantly with the number of neutrophils in BALF (Fig 4A–4C). Neutrophils are a major source of MMP-9 in inflamed lung [51]. Therefore, we suggest that the decrease in MMP-9, MMP-2 in BALF may result from the reduced number of neutrophils in BALF induced by Gal-9. Gal-9 reduced the level of MMP-9 secreted by neutrophils (Fig 6). These data suggested that Gal-9 involved in neutrophil-extracellular matrix interaction via the MMP-9 dependent pathway and regulates cell adhesion at multiple steps in part, both directly and indirectly. Further studies are needed to verify and clarify the role of MMP-9 in Gal-9-mediated activities.

In the present study, PPE-induced emphysema in Gal-9–deficient mice was significantly exacerbated as compared to that in WT mice (Fig 7A–7C). In addition, neutrophil infiltration in BALF in Gal-9-deficient mice was increased, although this difference was not statistically significant (Fig 7D). The levels of MMP-9, MMP-2 and TIMP-1 did not differ between WT and Gal-9-deficient mice 7 days after PPE treatment (Fig 7E). The reason for these gaps is hard to explain. We speculate that large amount of exogenously administered Gal-9 suppressed the migration of neutrophils and secretion of MMP-9 and MMP-2, but not endogenously released Gal-9 in WT mice. These data demonstrate that Gal-9-deficient mice are more susceptible to PPE-induced changes in the lung and that endogenously released Gal-9 has inhibitory effects in part on PPE-induced emphysema.

In conclusion, the present study demonstrates that Gal-9 prevents PPE-induced inflammation and emphysema in mice by inhibiting the infiltration of neutrophils, leading to reduced MMP-9 production. Although the precise mechanisms involved are still unclear, Gal-9-mediated inflammatory responses show beneficial effects in reducing PPE-induced emphysema, suggesting that Gal-9 could be a candidate therapeutic agent for the treatment of pulmonary emphysema and COPD.

## Supporting information

S1 Fig Representative photomicrographs of Diff-Quick stained cytospin slide of BALF. BAL cells stained with Diff-Quick solution after cytospin. Representative picture are shown on day 7 BALF from PPE (+) Gal-9 (-) (A) and PPE (+) Gal-9 (+) (B) at 400× magnification. Abbreviations: BAL, bronchoalveolar lavage; BALF, bronchoalveolar lavage fluid.
(TIF)
