# Supplementary figures and images for: Protective effect of Galectin-9 in murine model of lung emphysema: Involvement of neutrophil migration and MMP-9 production
Source: PLoS One. 2017 Jul 12;12(7):e0180742. doi: 10.1371/journal.pone.0180742 (PMC5507541; doi:10.1371/journal.pone.0180742)

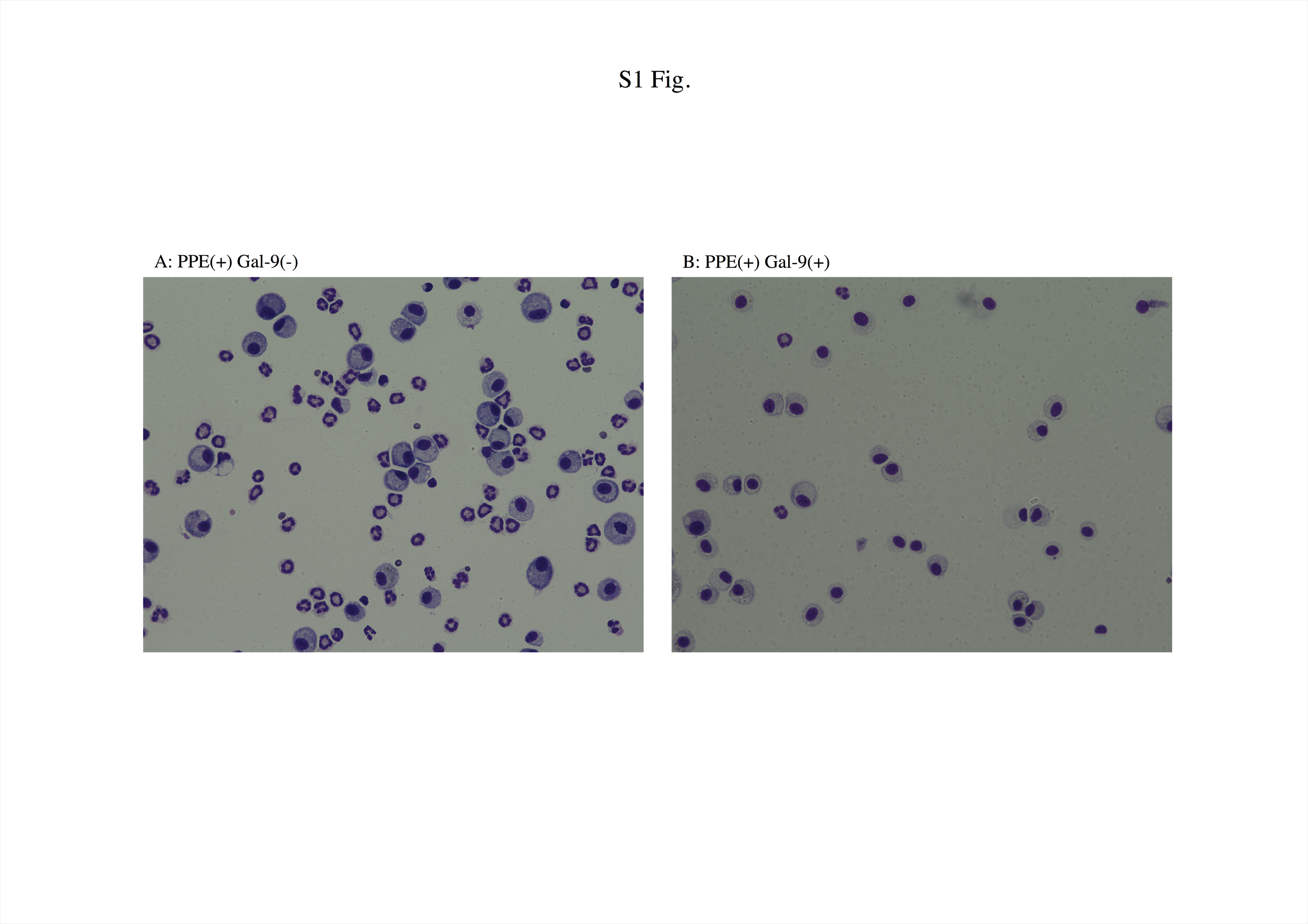

Supplement: S1 Fig — BAL cells stained with Diff-Quick solution after cytospin. Representative picture are shown on day 7 BALF from PPE (+) Gal-9 (-) (A) and PPE (+) Gal-9 (+) (B) at 400× magnification. Abbreviations: BAL, bronchoalveolar lavage; BALF, bronchoalveolar lavage fluid. (TIF) [file pone.0180742.s001.tif]
